# Supplementary material for: The cost-effectiveness of MDMA-assisted psychotherapy for the treatment of chronic, treatment-resistant PTSD
Source: PLoS One. 2020 Oct 14;15(10):e0239997. doi: 10.1371/journal.pone.0239997 (PMC7556534; doi:10.1371/journal.pone.0239997)
Supplement: S1 File — (DOCX) [file pone.0239997.s001.docx]

**Table of Contents**

[**Alternative comparison: Phase 2 trial active group versus control group at primary endpoint** 1](#_Toc45201090)

[**Estimation of intervention costs** 3](#_Toc45201091)

[**Estimation of PTSD-related medical care costs.** 3](#_Toc45201092)

[**Further evidence of MAP effectiveness** 6](#_Toc45201093)

[**References** 7](#_Toc45201094)

# **Alternative comparison: Phase 2 trial active group versus control group at primary endpoint**

Because the control condition does not represent a viable treatment for PTSD, the primary analysis compares the active MDMA group at baseline and at follow-up. See Supplementary Figure 1.


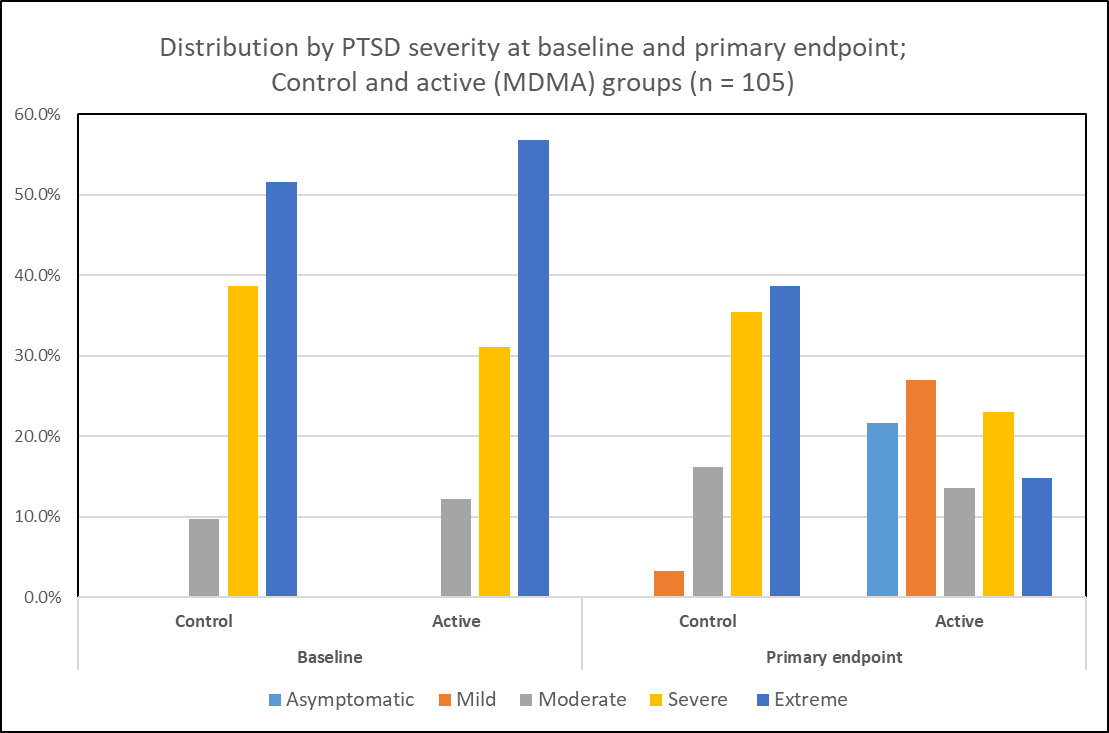


**Supplementary Figure 1: Distribution of phase 2 trial subjects by severity.**

*Legend*. Patients classified according to global CAPS-IV score: 0-19: asymptomatic/few; 20-39: mild PTSD/subthreshold; 40-59: moderate PTSD/threshold; 60-79=severe, >=80 extreme PTSD symptomatology(1).

However, in an exploratory analysis we compared the control group with the active group at follow-up. In that scenario, the difference in cost is that of the MDMA itself, since the control group otherwise received precisely the same protocol as the active group. The difference in effectiveness reflects the reduction in CAPS scores associated with the control condition. Thus, compared with the primary analysis, both the incremental effectiveness and the incremental costs of MAP are smaller. As shown in Supplementary Table 1, for a cohort of 1,000 patients, MAP is associated with 3,985 incremental discounted QALYs and savings of $78 million over a 30-year horizon.

**Supplementary Table 1: Discounted cost, health benefits and cost-effectiveness outcomes in exploratory comparison of active and control group at primary endpoint.**

*Legend.* 1,000 hypothetical subjects; 30-year analytic horizon.

# **Estimation of intervention costs**

To obtain unit costs, CPT codes were assigned to each MAP activity. These include CPT code 90837 for psychotherapy, pro-rated over the 90 minute length of the ‘pre’ and ‘post’ sessions and the 8 hours of the MDMA sessions; CPT codes 96130 and 90792 for intake and screening respectively; and, for the 10% of patients who, due to potential cardiac risk identified at screening required nuclear stress tests and carotid ultrasound, 93015 and 93880 respectively. Two therapists participated in all sessions. The cost associated with each CPT codes 009837, 96130, and 90792 was assigned the average cost of eight metropolitan areas as provided by “FAIR Health Consumer” which maintains a large private and public payer national claims data base^(2)^; or, in the case of 93015 and 93880, the Medicare allowable reimbursement amount as provided by the Centers for Medicare and Medicaid Services^(3)^. As reported by MAPS’ accounting data, $177 per session was spent for the MDMA itself, $121 for tests for pregnancy and drugs of abuse at screening, or $282 per patient completing the MAP protocol.

# **Estimation of PTSD-related medical care costs.**

We identified six empirical studies of medical care costs for populations with PTSD in the U. S. We initially excluded studies that addressed special populations, e.g. those with co-morbid psychotic disorders; or reported utilization without associated costs. We qualitatively rated each study according to how closely its characteristics matched participants in the MAPS trials. Ivanova 2011 was a large study (N = 9,114 and 9,720 for Medicaid and private insurance claims databases, respectively) that separately estimated costs for Medicaid and for private insurance. We treated this as two studies. We then weighted each study by the square root of its sample size (representing statistical variance) to calculate a mean annual expenditure of $18,044. After omitting Walker 2005 (due to its implausibly low estimate of $4,234), and Meyers 2013 (omits general medical costs) we arrived at $19,888. Since none of these studies reported the distribution of subjects by PTSD severity, we conservatively (unfavorable to cost-effectiveness) assumed that this annual cost was associated with severe PTSD. For costs of asymptomatic PTSD we adopted the $4,946 (adjusted to 2019 dollars) estimate from Ivanovna 2011 (patients randomly selected from a large private insurance database)(4). We then adjusted the $19,888 cost across mild - severe severity categories to align with the mild-to-severe 2.04 ratio of costs found in a study of 1,225 female members of an HMO(5). We conducted sensitivity analyses on these assumptions.

**Supplementary Table 2. Qualitative review of studies reporting cost of medical care for people with PTSD.**

*Legend.* Green, yellow, red signify good, neutral and poor (respectively) representation of CEA target population per MAPS 6 Phase 2 trials

**Supplementary Table 3. Mean annual costs of medical care for severe PTSD.**

*Legend.* These estimates are based on the figures shown in Table 2.

**
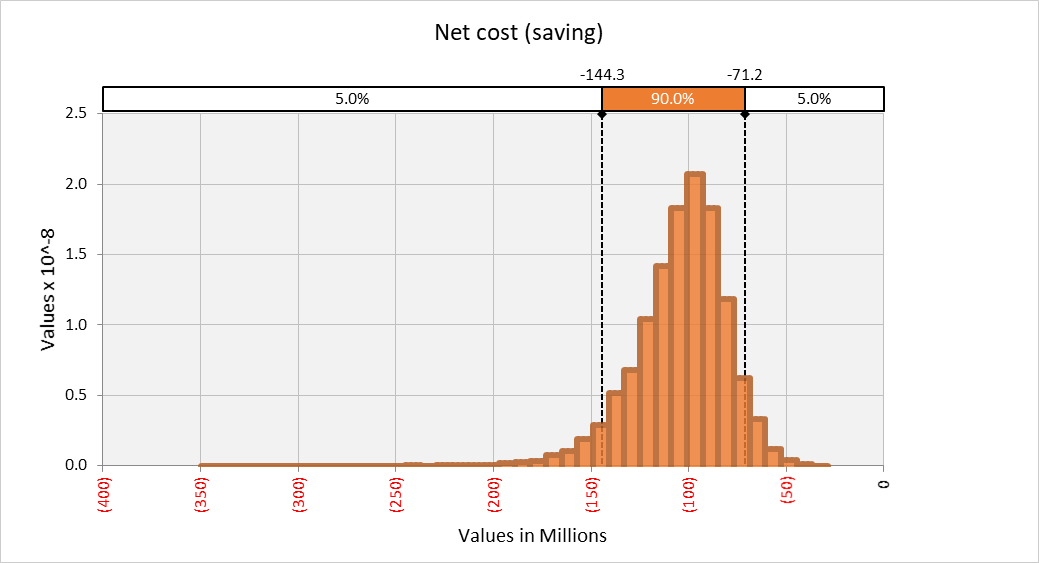
**

**Supplementary Figure 2. Multivariable sensitivity analysis: Net discounted cost per 1,000 patients projected over 30 years.**

*Legend****.*** Outcome of 10,000 iterations of a Monte Carlo simulations (probabilistic sensitivity analysis) in which all input variables are varied simultaneously based on distributions show in Table 1 of main text.

**
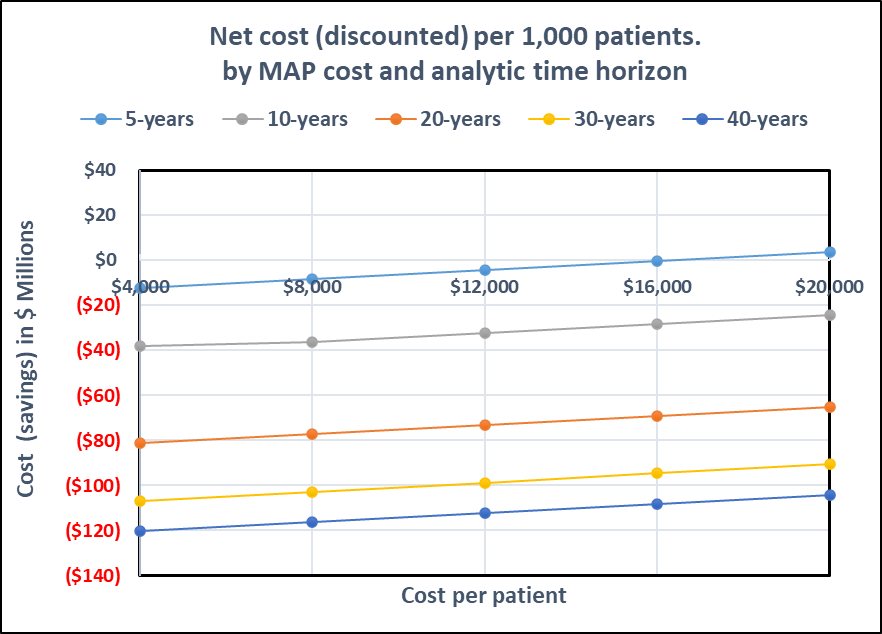
**

**Supplementary Figure 3. Two-way Sensitivity analysis: Net discounted cost per 1,000 patients versus cost of MAP and time horizon.**

*Legend.* Net costs of MAP where cost of MAP varies from $4,000 - $16,000 (base case $7,543) and analytic time horizon varies from 5 to 40 years (base case 30 years)

# **Further evidence of MAP effectiveness**

On May 12, 2020, MAPS announced the results of an interim analysis of one of two of its Phase 3 clinical trials of MAP to treat PTSD. “The analysis was conducted by an independent Data Monitoring Committee, which reviewed the results from the first 60 out of 100 participants. The analysis revealed a 90% or greater probability that the trial will detect statistically significant results when all participants have been treated, and that the trial will not require additional participants beyond the first 100. The interim analysis was approved by the FDA as part of MAPS’ Statistical Analysis Plan approved by the FDA.”(6)

The primary endpoint of the MAPS-funded efficacy studies is a checklist-based instrument, the CAPS-IV scale that measures the presence and severity PTSD. However, there are aspects of mental health-related well-being that cannot be captured by any single quantitative measure. In the context of a small (N=19) long-term follow-up study, subjects responding to a semi-structured qualitative interview reported positive changes in such varied dimensions as improved relationships and social skills: reduced medication and substance abuse; openness to future therapies and improved self-awareness. Even clients who only improved modestly as reflected in their CAPS-IV scores reported substantial positive outcomes on these other qualitative dimensions.(7)

A recent study of long term outcomes of 83 patients from the six MAPS phase 2 trials, found that between study exit and at least 12 months follow-up, the percentage of subjects who no longer met criteria for PTSD fell from 56% to 67% reflecting the mean decrease in CAPS-IV score in these subjects of 5.2 points, *p* = 0.0245.(8) These patients also reported benefits at 12-months follow-up in an open-ended questionnaire. 97.6% reported benefit, and among those, 92.2% stated that these benefits were lasting. 53.2% indicated large benefits that lasted or continued to grow.

# **References**

1. Weathers FW, Keane TM, Davidson JR. Clinician-administered PTSD scale: a review of the first ten years of research. Depression and anxiety. 2001;13(3):132-56.

2. FAIR Health Consumer. Search for medical and hospital costs 2020 [Available from: <https://www.fairhealthconsumer.org/>.

3. Centers for Medicaid and Medicare Services. Physician Fee Schedule Search, 2020 [Available from: <https://www.cms.gov/apps/physician-fee-schedule/license-agreement.aspx>.

4. Greenberg P, Corey-Lisle PK, Birnbaum H, Marynchenko M, Claxton A. Economic implications of treatment-resistant depression among employees. PharmacoEconomics. 2004;22(6):363-73.

5. Walker EA, Katon W, Russo J, Ciechanowski P, Newman E, Wagner AW. Health care costs associated with posttraumatic stress disorder symptoms in women. Arch Gen Psychiatry. 2003;60(4):369-74.

6. Interim Analysis Shows At Least 90% Chance of Statistically Significant Difference in PTSD Symptoms after MDMA-assisted Psychotherapy [press release]. May 12, 2020.

7. Barone W, Beck J, Mitsunaga-Whitten M, Perl P. Perceived Benefits of MDMA-Assisted Psychotherapy beyond Symptom Reduction: Qualitative Follow-Up Study of a Clinical Trial for Individuals with Treatment-Resistant PTSD. J Psychoactive Drugs. 2019;51(2):199-208.

8. Jerome L, Feduccia AA, Wang JB, Hamilton S, Yazar-Klosinski B, Emerson A, et al. Long-term follow-up outcomes of MDMA-assisted psychotherapy for treatment of PTSD: a longitudinal pooled analysis of six phase 2 trials. Psychopharmacology (Berl). 2020.
